# Ubiquitous Neocortical Decoding of Tactile Input Patterns

**DOI:** 10.3389/fncel.2019.00140

**Published:** 2019-04-12

**Authors:** Jonas M. D. Enander, Anton Spanne, Alberto Mazzoni, Fredrik Bengtsson, Calogero Maria Oddo, Henrik Jörntell

**Affiliations:** ^1^Neural Basis of Sensorimotor Control, Department of Experimental Medical Science, Lund University, Lund, Sweden; ^2^The BioRobotics Institute, Scuola Superiore Sant’Anna, Pisa, Italy

**Keywords:** tactile, sensory, neuron, neurophysiology, neocortex, spike responses

## Abstract

Whereas functional localization historically has been a key concept in neuroscience, direct neuronal recordings show that input of a particular modality can be recorded well outside its primary receiving areas in the neocortex. Here, we wanted to explore if such spatially unbounded inputs potentially contain any information about the quality of the input received. We utilized a recently introduced approach to study the neuronal decoding capacity at a high resolution by delivering a set of electrical, highly reproducible spatiotemporal tactile afferent activation patterns to the skin of the contralateral second digit of the forepaw of the anesthetized rat. Surprisingly, we found that neurons in all areas recorded from, across all cortical depths tested, could decode the tactile input patterns, including neurons of the primary visual cortex. Within both somatosensory and visual cortical areas, the combined decoding accuracy of a population of neurons was higher than for the best performing single neuron within the respective area. Such cooperative decoding indicates that not only did individual neurons decode the input, they also did so by generating responses with different temporal profiles compared to other neurons, which suggests that each neuron could have unique contributions to the tactile information processing. These findings suggest that tactile processing in principle could be globally distributed in the neocortex, possibly for comparison with internal expectations and disambiguation processes relying on other modalities.

## Introduction

Studies using global network analysis with non-invasive methods in humans indicate that the neocortex is functionally heavily interconnected (Bullmore and Sporns, 2009), suggesting that any information available to one area of the neocortex could also be available to many other areas. In contrast, the idea of functional localization advocates that each area of the neocortex has an innate specificity of function and that information of a specific modality or a specific combination of modalities would be processed solely or predominantly in a localized area. The latter line of thought has a long history (Broca, 1861; Penfield and Boldrey, 1937) and has seen positive results when studied with functional magnetic resonance imaging (fMRI; Maldjian et al., 1999), electrocorticogram (ECoG), electroencephalogram (EEG; Baumgartner et al., 1992) and single cell recordings (Kaas et al., 1979). Of the two lines of ideas, functional localization has perhaps the strongest and widest presence in the field of neuroscience and is often regarded as fundamental to brain function both in science and in the clinic (Desmurget and Sirigu, 2015; Marshall and Meltzoff, 2015). But even at the time of its conception, many theorists raised concerns that the neocortex could be a more globally integrated system where functional localization may have limited explanatory potential (Andral, 1833; Brown-Séquard, 1877; Prince, 1910; Lashley, 1929). Moreover, in recent years clinicians have started to question “commonly held assumptions underlying presumed correlations between particular lesion locations and the associated behavioral deficits” (Sathian and Crosson, 2015) and there are for example clinical findings that unilateral cortical lesions cause bilateral tactile sensory deficits (Brasil-Neto and de Lima, 2008) and that parietal stroke can affect tactile sensation (Bassetti et al., 1993).

A number of studies have presented empirical evidence that basal sensorimotor signals can be found in widespread areas of the neocortex. These studies include observations of spatially unbounded cortical distribution of inputs related to a specific modality (Fu et al., 2003; Ferezou et al., 2007; Frostig et al., 2008; Hihara et al., 2015; Rancz et al., 2015) or behavioral modulation, i.e., locomotor-related signals in primary visual cortex (Keller et al., 2012; Saleem et al., 2013). The multisensory influences in presumptive unimodal sensory areas are so pervasive that it has been suggested that the neocortex is essentially multisensory (Ghazanfar and Schroeder, 2006). However, so far these studies focused on the binary question if unbounded sensory input is present. The question of what such unbounded neural activity represents, in terms of input quality and information quantity, has not been studied.

In order to be able to quantify whether the activity of neurons carries any information regarding the “what” component of external input, one needs a number of diversified inputs that each has a high degree of reproducibility. We previously introduced a method to deliver such reproducible and diversified spatiotemporal input patterns by electrical activation of tactile afferents in local digit skin and showed that cells in the primary somatosensory cortex (S1) of the rat are capable of decoding these tactile inputs with high accuracy (Oddo et al., 2017). Using this approach, we recently reported that the neurons of the S1 cortex have access to information about the “what” component of ipsilateral tactile inputs, just like they have for contralateral inputs (Genna et al., 2018). Here, we show that in non-paw S1 regions and in non-S1 regions across the dorsal neocortical surface, including within visual cortical areas, the responses of individual neocortical neurons contain information about the “what” component of tactile inputs to the second digit of the forepaw.

## Materials and Methods

### Surgical Procedures

Adult Sprague-Dawley rats (*N* = 18, male sex, weight 306–420 g) were prepared and maintained under anesthesia with a ketamine (100 mg/ml) and xylazine (20 mg/ml) mixture. Prior to the induction of the anesthesia, the animals were sedated with isoflurane (3% mixed with air for 60–120 s). Anesthesia was induced with an intraperitoneal injection (Ketamine: xylazine concentration ratio of 15:1, 1.5 ml/kg) and further maintained with a continuous infusion through an intravenous catheter inserted into the right femoral vein (concentration ratio of 20:1, approximately 5 mg/kg ketamine per hour). The absence of withdrawal reflexes to noxious pinch to the hind paw was used to characterize adequate anesthesia. The duration of the experiments did not exceed 8 h, after which the animals were sacrificed.

The use of anesthesia was motivated by that we needed to make sure that the mechanical stability of the brain was consistently high throughout the experiments in order to run the long-term *in vivo* patch clamp recordings in loose-patch, cell-attached, mode required to achieve a high number of repetitions of the stimuli used (see below). Ketamine/xylazine anesthesia has previously been shown to not affect the order of neuronal recruitment of a sheet of layer 5 neurons in spontaneous brain activity fluctuations and evoked responses as compared to the awake condition, suggesting that the neocortical network may work close to normal (Luczak and Barthó, 2012; Bermudez Contreras et al., 2013). Exactly how the anesthetic effect on consciousness is achieved is an open question, but may depend on a generally lower network activity (Constantinople and Bruno, 2011) and dissociative effects on the scale of seconds rather than milliseconds.

The craniectomy of the right hemicranium extended from a reference point located 1 mm rostral and 2 mm lateral to the bregma to approximately 9 mm caudally and 5 mm laterally relative to the same reference point (Paxinos and Watson, 2006). Hence, the exposure included the primary motor cortex, the primary somatosensory cortex and the rostral part of the primary visual cortex. During the control experiments with a reduced audiovisual background (described below) the craniectomy extended 3 mm laterally to the reference point described above and between 5 and 8 mm caudally to bregma. This exposure was thus centered on the primary visual cortex. An ECoG-electrode was placed on the surface of the cortex at the rostral end of the craniectomy. For recording stability, a cap of agarose (0.03 g/ml dissolved in physiological saline) was made to cover the exposed part of the brain. The exposed part of the brain was inspected with a microscope during both insertion and extraction of the recording electrodes. The state of the rat was continuously evaluated based on skin tone, respiration rate and the ECoG signal. The ECoG signal was monitored for occurrences of sleep spindles, which occurred irregularly, thus indicating deep sleep (Niedermeyer and da Silva, 2005).

### Recordings

**Figure 1:**
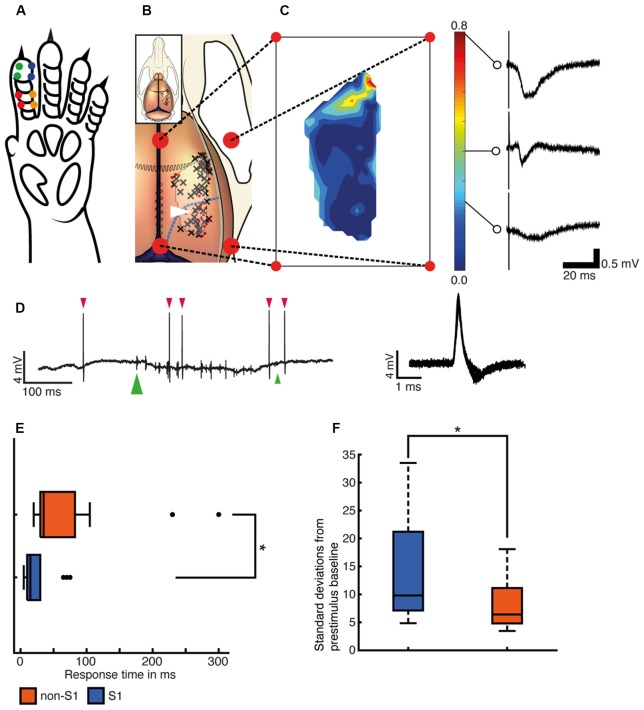
Recording sites and responses to single pulse stimulations. **(A)** Illustration of the left forepaw of the rat to indicate the locations of needle electrode pairs (colored dots) used for the delivery of the tactile afferent stimuli. **(B)** Illustration of the rat brain and skull. Black crosses (*N* = 45) indicate electrode tracks/recording sites with at least one neuron with valid decoding of the tactile input patterns (see text) and red dots (*N* = 33) indicate sites without any such neurons. Blue dashed line indicates the border of the visual cortical regions (Papathanasiou et al., 2006; Paxinos and Watson, 2006). The outline of the contour plot in panel **(C)** is indicated as a lighter area with a dark border for reference. **(C)** Contour plot of the amplitude of the local field potentials evoked using single pulse stimulation in each of the recording sites. The color calibration bar is shown together with example sensory-evoked local field potentials (SE-LFPs) for three different amplitudes. **(D)** Left, an example of a raw spike recording during the presentation of stimulation pattern F5. Neuronal spikes are marked with red arrowhead above. The onset of the stimulation pattern is marked with a green large arrowhead below and its termination time is marked with a small green arrowhead. Right, 60 spikes from the example recording are shown superimposed, centered on peak amplitude. Note that the patch clamp electrode allows highly isolated recordings very close to single neurons, which typically results in large spike amplitudes and somewhat wider spikes than with metal microelectrodes. **(E)** Distribution of the onset latency times for the spike responses evoked by the stimulation patterns (S1 *N* = 26, non-S1 *N* = 13). Asterisk indicates that the differences were statistically significant (two-sided Wilcoxon rank sum test, *p* = 5.5051e-04). **(F)** Maximal amplitudes for spike responses evoked by the stimulation patterns indicated as multiples of the standard deviation (SD) of the baseline activity (S1 *N* = 26, non-S1 *N* = 13). Two outliers in the “somatosensory” group (at 52.8 and 148.4 SDs) were not included to facilitate comparison between the two groups.

All recordings were made *in vivo* in the right hemisphere. In most experiments, at least one recording was made from the forepaw region of the primary somatosensory cortex, as estimated by the focus of local field potentials evoked by electrical stimulation of digit 2 on the left forepaw (Figure 1A). This was made to verify that the delivered stimuli worked. Additional recording sites were chosen by the experimenter to cover a wide extent of the exposed brain as possible and included also parts of the S1 that were outside its forepaw region. A photo was taken of the exposed brain and the location of each recording site was indicated on this photo. Each recording site was subsequently indicated on a common picture of a rat brain based on anatomical landmarks and distances (Figure 1B).

Individual neurons were recorded with patch clamp pipettes extracellularly in the loose-patch current clamp recording mode. Patch clamp pipettes were pulled from borosilicate glass capillaries to 10–30 MΩ using a Sutter Instruments (Novato, CA, USA) P-97 horizontal puller, and back-filled with an electrolyte solution. The composition of the electrolyte solution in the patch pipettes was (in mM) potassium-gluconate (135), HEPES (10), KCl (6.0), Mg-ATP (2), EGTA (10). The solution was titrated to 7.35–7.40 pH using 1 M KOH. During the slow advancement of the recording electrode (approximately 0.002 mm/s) with an electrical stepping motor, all four skin stimulation sites were activated synchronously at a rate of one pulse per second. Any neuron encountered was recorded and in some cases, a number of neurons were recorded in sequence in the same electrode track. In most experiments, the recorded signal was output on a loudspeaker at the same time as it was displayed on an LCD computer screen for monitoring of the signal by the experimenter. The screens were therefore at a remote location to the animal (>2 m away on the lateral side) in an otherwise normally lit room with a humming background noise from fans in the electrical equipment. To verify that these circumstances were not a factor defining the results of the decoding analysis, we made a set of control experiments (*N* = 2 animals and *N* = 16 neurons in the visual cortex) where the loudspeakers were turned off and the animals were visually shielded by screens located approximately 150 mm lateral to the eyes.

The recording depths from the surface of the brain were saved for all neurons recorded. All data was digitized at 100 KHz using CED 1401 mk2 hardware and Spike2 software (Cambridge Electronic Design, CED, Cambridge, UK). Spikes were identified using in-house software based on template matching. All spike detection was carefully controlled by the visual inspection of zoomed-in raw data traces throughout the stored recording.

### Stimulation

Four pairs of intracutaneous needle electrodes inserted into predefined sites in the skin on the volar side of digit 2 of the left forepaw (Figure 1A). The inter-needle distance was 2–3 mm in each pair. For each skin site, the stimulation pulse was set to an intensity of 0.5 mA with a duration of 0.14 ms (DS3 Isolated Stimulator, Digitimer, UK), which is 2.5 times greater than the threshold for activating tactile afferents using this approach (Rasmusson and Northgrave, 1997; Bengtsson et al., 2013) but well below the threshold intensity where A-delta and C-fibers start to become recruited (peak activation requires 6–10 times threshold intensity; Ekerot et al., 1987).

**Figure 2:**
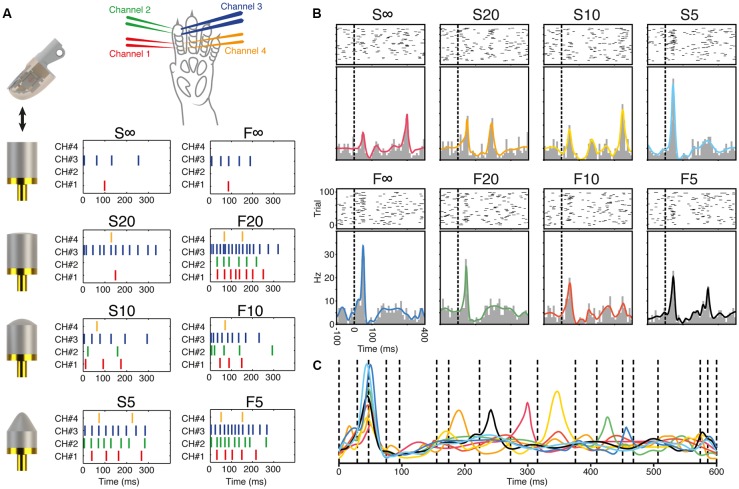
Generation of the stimulation patterns and responses of a sample V1 neuron. **(A)** Illustration of the sensorized artificial fingertip and the objects it was dynamically moved against to generate the stimulation patterns. The figure also shows the location of the stimulation channels on the rat digit skin and the eight spatiotemporal stimulation patterns used to stimulate the digit skin. **(B)** Raster plots, peristimulus histograms and kernel density estimation (KDE) curves for the responses generated by the eight different stimulation patterns in a sample neuron recorded in V1 cortex. **(C)** The eight KDE response curves of the sample neuron are superimposed and shown together with the neuron-specific defined time windows used in the subsequent decoding analysis.

Through this electrical interface, eight predefined spatiotemporal patterns of skin activation were delivered (Figure 2A, the stimulation patterns are indicated as F5, S5, F10, S10, F20, S20, F∞ and S∞). These stimulation patterns lasted less than 350 ms and consecutive deliveries were separated by 1.8 s. Additionally, for each skin stimulation site, repeated single pulse stimulation trains of five consecutive pulses separated by 333 ms were also delivered. The spatiotemporal patterns and single pulse stimulation trains were presented repeatedly up to 100 times in a pseudo-random order (repeated randomized order). The patterns were exactly the same as in the article of Oddo et al. (2017).

### Generation of the Spatiotemporal Stimulation Patterns

The process underlying the generation of the spatiotemporal stimulation patterns delivered to the electrical interface on the second digit of the rat has been described in detail previously (Oddo et al., 2017). Briefly, we used an artificial fingertip equipped with a set of neuromorphic sensors to transduce a set of tactile events. The tactile events consisted of dynamic indentations of the sensorized fingertip skin against a set of predefined shapes using a sinusoidal one-dimensional motion controlled by a cyclic motor (Figure 2A). The core element of the sensorized fingertip was a Micro Electro Mechanical System (MEMS) sensor with four transducing piezoresistors implanted at the base of a cross-shaped structure. The MEMS was packaged with polymeric compliant material (Dragon Skin, Smooth-On, Macungie, PA, USA). MEMS data were sampled at 380 Hz per sensor output by a 24-bit Analog to Digital Converter (ADS1258, Texas Instruments, Dallas, TX, USA) integrated at the fingertip, and acquired *via* SPI by a Field Programmable Gate Array (Cyclone II FPGA, Altera, USA). The FPGA acquired the information, which would correspond to the receptor potentials of skin sensors (Woo et al., 2015). These “receptor potentials” were converted to spike trains by our neuromorphic artificial touch system which uses a customized implementation of Izhikevich spiking neuron model. The spiking neuron model was originally designed to emulate two artificial mechanoreceptor types mimicking slow (S-type) and fast (F-type) adapting receptors. The resulting eight spatiotemporal patterns should be regarded as eight different types of skin-object interactions. The four needle electrode pairs of the interface were 1-to-1 related to the four neuromorphic sensors of the artificial fingertip.

As shown previously (Oddo et al., 2017), with the type of dynamic indentation movement used, available evidence indicates that there is in principle little difference in the spike activation between slowly and rapidly adapting tactile mechanoreceptors (Johansson et al., 1982; Jenmalm et al., 2003). Hence, the artificial fingertip allowed us to synthesize spatiotemporal patterns of skin sensor activation at quasi-natural rates that follow a similar overall temporal modulation, or “envelope” (Middleton et al., 2006), of activation as biological skin sensors display under dynamic indentation.

## Statistical Analysis

### Local Field Potentials

In addition to the neuronal recordings, we analyzed the sensory evoked local field potential (SE-LFP) responses. SE-LFP data was obtained using single pulse stimulation of the four intracutaneous stimulation sites individually, each repeated up to 100 times as described above. All the responses evoked from each skin site were first superimposed. In order to minimize the impact of evoked spikes on the SE-LFP analysis, we then removed 50% outliers for each sample time point. The normal distribution of the amplitude of the remaining raw data was calculated for a pretrigger period of 100 ms. An SE-LFP was assumed to be evoked if the average post-trigger signal within 100 ms after the onset of the stimulation reached below 5 standard deviations (SDs) relative to the pretrigger baseline. The average signal was filtered through a 10-order one-dimensional median filter (moving average). From this filtered average signal, an automatic detection method was used to identify the response latency time (negative crossing of the −2 SD), duration (time until positive recrossing of the −2 SD line) and amplitude of all SE-LFPs. The amplitude of the SE-LFP was the minimum value inside the defined response (as all recordings were below 0.292 mm of depth in the cortex, all local SE-LFPs were assumed to be negative).

The largest SE-LFP amplitude at each recording site was used to create a contour plot as a topographic visualization of the SE-LFP distribution (Figure 1C).

As the stimulation patterns lasted up to 350 ms, there was a chance that they evoked weaker, less frequent LFPs at irregular latency times than what could be detected by the single pulse stimulation above. We, therefore, evaluated also the LFPs evoked by the individual stimulation patterns. From each recorded neuron (*N* = 116) and for each stimulus presentation (eight patterns, 50–100 presentations each), we measured the LFP activity in two contiguous periods of recording, a 400 ms pretrigger and a 400 ms post-trigger period, respectively. The signal was first low-pass filtered by performing a rolling boxcar mean of 10 ms, followed by a resampling from 100 kHz to 1 kHz and by application of a first-order butterworth bandpass filter (50–499 Hz). The DC offset of each recorded response was removed by subtracting the median voltage value of its pretrigger period. From the population of responses for each individual neuron recording, the mean and SD of the baseline were calculated from the 400 ms pre-trigger period. An LFP was defined as a drop in the voltage signal of at least 2 SDs below baseline for at least 10 ms. The onset, duration and amplitude of the LFPs were analyzed separately for pre- and post-trigger periods. LFPs that began in the pre-trigger period and continued into the post-trigger period was counted as a pre-trigger event.

Differences in the distributions of the net number of LFPs, their durations and amplitudes for pre- vs. post-trigger periods were analyzed using Wilcoxon signed-rank test. To capture if there was any time-dependent difference between the pre- and post-trigger periods, the raw signal was replaced with a signal that solely contained the calculated onsets, durations and amplitudes of the LFPs. Hence, in this signal, at each LFP onset, a boxcar deviation was added with a height that corresponded to the LFP amplitude and a duration that equaled the calculated LFP duration. The boxcar deviations were then used to calculate the area under the curve (AUC) for each LFP.

### Response Latencies and Response Intensities

To obtain an estimate of the onset latency for evoked spike activity, we generated peristimulus spike frequency time histograms (PSTHs), with a bin size of 5 ms, both for responses evoked by the spatiotemporal patterns and for the responses evoked by the single pulse stimulations. We pooled the data for the spatiotemporal patterns and the single pulse stimulations, respectively, since we were not interested in internal differences between different patterns or stimulation sites for this analysis. The analysis of the responses evoked by the spatiotemporal pattern included data from a time period of 500 ms pretrigger and 500 ms post-trigger (the trigger was defined as the stimulus onset). The single pulse stimulations included a time period from 100 ms pretrigger to 100 ms post-trigger. From the pretrigger period, we calculated the mean and SD of the baseline spike frequency. For the post-trigger period, onset latency was defined as first encountered histogram bar in a series of at least two consecutive bars exceeding three SDs from the baseline. A two-sided Wilcoxon rank sum test was used to test for significant differences between data recorded in S1 and in non-S1.

To estimate the intensity of the excitatory responses, we calculated the maximal spike frequency change from baseline in a period starting at the onset latency time and reaching until the end of the post-trigger period. The response intensity was measured as the number of SDs from the pretrigger baseline. A two-sided Wilcoxon rank sum test was used to test for significant differences between data recorded in S1 and non-S1.

### Representation of the Average Evoked Responses as a Continuous Function

To generate a better representation of the evoked spike responses than provided by traditional PSTHs, we transformed the spike times to a spike density function [i.e., a form of kernel density estimation (KDE)]. For each neuron, the individual spike responses were grouped by stimulation pattern and the corresponding KDE for each group, or stimulation pattern, was calculated using the solution of Shimazaki and Shinomoto (2010). KDE provides a more accurate representation of the spike time data than the PSTH since it avoids the loss of information associated with binning. Here, we used it also as a means to transform the spike responses into time-continuous functions, one function for each stimulation pattern and neuron (Figures 2B,C), which were necessary for the decoding analysis.

### Decoding Analysis

The aim of the decoding analysis was to obtain a quantitative measure of the degree by which the responses evoked by repeated applications of one stimulation pattern differed from the responses evoked by the other stimulation patterns. As individual responses contained episodes of increased spiking activity, visible as peaks of activity in the PSTHs and the KDEs (Figures 2B,C), we developed a method to automatically identify such densifications of spiking activity. The KDEs for each of the eight stimulation patterns were superimposed and the max value at each time point (at 1 ms resolution) was used to generate a single compound density function. In this compound density function, each local minimum, which typically signified a boundary between two consecutive peaks, was identified and used as a time boundary. Thus, the full duration of the compound density function was segmented into bins or time windows. For each time window, the AUC was calculated for the compound density function. For the AUC calculation, the baseline was the lowest value that occurred within the time window. The AUCs of each time window was subsequently normalized to the largest AUC of all of the time windows. Any time window with an AUC less than 2.5% of the largest AUC was excluded from further analysis.

In a subset of the neurons, the peaks in the KDE curves were too weak for this type of analysis to be performed. We set a threshold criterion of at least three peaks (across the KDEs of all eight stimulation patterns) with an amplitude exceeding 200% of the baseline of the KDE (100%) within 50 ms of the peak’s deviation from the baseline. Neurons that did not contain this minimum of peak responses were excluded from further analysis using this approach.

In the next step, all the raw spike responses of evoked by each stimulation pattern were randomly split into a training set and a test set (50% of the responses in each set). Each spike response was transformed into a continuous signal by convolving each spike with an exponential kernel with a time constant of 5 ms. Both training and test responses were subsequently expanded by recombination, which resulted in the generation of a higher number of combined responses than in the original recording data (the recombination or “bootstrapping” procedure is described in detail below). Each combined response was segmented by the time windows defined as described above. For each time window, the AUC of the combined response was computed. The values for each time window were normalized against the largest AUC measured for that time window in all of the combined responses for the respective set (training or test set). Each time window defined was considered a unique response dimension, and the normalized AUC of the combined response was the scalar value for that response in the respective dimension. The position of each combined response in high dimensional space could hence be defined. Consequently, the Euclidean distance to all other combined responses could be calculated. For each combined response of the test set, their distances to the combined responses of the training set were calculated. For this purpose, we used a k-Nearest Neighbor (kNN) classification algorithm for the nine nearest neighbors (the nine closest responses in the high-dimensional space) as the basis for the classification of the test set, where the classification was made against the training set. If a majority of the closest responses were generated by the same stimulation pattern as the one generating the test response, the classification of that response was correct. Based on the results of the kNN analysis across the population of analyzed responses (i.e., the test sets for each of the eight stimulation patterns), we constructed confusion matrices, which indicates the percentage of the correctly classified responses as well as the percentage of the confusing stimulation pattern in cases of incorrect classification, and calculated the decoding accuracy (mean decoding performance). The procedure in this paragraph was iterated for 50 times, each time with a new random split of the raw data into a training set and test set and a new bootstrapping process. The confusion matrices are shown, and the mean decoding performances reported, represent the average of these 50 iterations.

### Shuffled Control Decoding

The theoretical chance level of the decoding performance when performing a kNN-classification analysis depends on the number of classes. The chance level for correct classification when there are two classes is 50%, i.e., 100% divided by the number of classes. In our setting where we had eight classes or spatiotemporal tactile stimulation patterns, the theoretical chance level was hence 12.5% (100%/8). For each neuron, we also tested the effect of shuffling the responses with respect to the stimulation pattern. This test was done to provide an internal control for each neuron, i.e., if there were features in the neuronal firing patterns, or in the method, that would bias neurons to not report chance decoding level (12.5%) when the analyzed responses were independent of the stimulation pattern. This control decoding analysis (referred to as “shuffled control decoding” in the Results) worked exactly as the decoding analysis described previously, but prior to each data split into a test and training set the labels for each stimulation pattern were shuffled.

### Confusion Matrices for Analysis of Response Differences Between Neurons

In some cases, we also compared the responses generated by different neurons to the same stimulation pattern (this was done for four or five neurons at a time). We used the same approach as above, but the response groups, in this case, consisted of the raw responses generated by the different neurons to the same stimulation pattern.

### Cooperative Decoding Between Neurons

In order to analyze the additional decoding capacity that could be provided by a population of neurons, we extended the analysis above into a “cooperative” decoding analysis. This was a similar approach as in Oddo et al. (2017) rather than principal component analysis (PCA), here we instead used the time window response segmentation as the basic analysis approach. To illustrate how decoding depended on the duration of the response and the number of neurons taken into account, this analysis aimed at finding the best possible combination of neurons for each response duration. For each given time interval/duration analyzed, an iteration through the available neurons was performed in which we calculated their decoding within that time interval. In that time interval, each neuron had its own set of time windows/bins, thus giving the neuron a response dimensionality M_i_ (one dimension per time window). The neuron with the best decoding [a] was kept. In the next iteration, the remaining neurons were scanned and the kNN decoding analysis was performed with the dimensionality M_a_ + M_i_. The best combination was stored [a, b], and the procedure was repeated again until the maximum number of neurons (arbitrarily defined as 10 in our case) had been reached. Thus, the dimensionality increased by the number of time windows defined for each added neuron. The procedure was repeated for all time intervals/durations, as indicated (Figure 4B) and was performed for S1 neurons and neurons recorded in the visual cortex, respectively.

**Figure 3:**
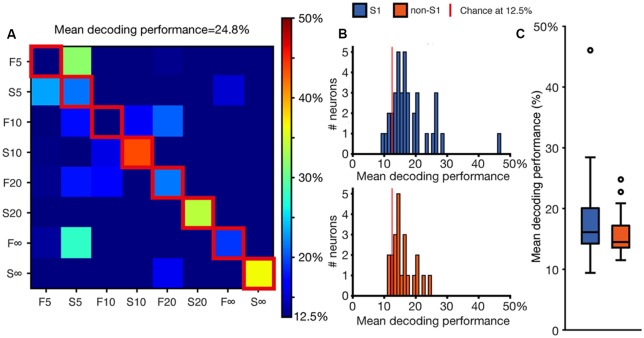
Decoding performance at the single neuron level. **(A)** A confusion matrix illustrating the degree of specificity of the spike responses to the different stimulation patterns for the sample neuron also shown in Figure 2. **(B)** Mean decoding performance across the population of neurons. Chance decoding level is at 12.5% (for the comparison between eight stimulation patterns) as indicated by the red vertical line. **(C)** Box plot of the mean decoding performance for S1 (*N* = 44) and non-S1 (*N* = 24) neurons, respectively.

**Figure 4:**
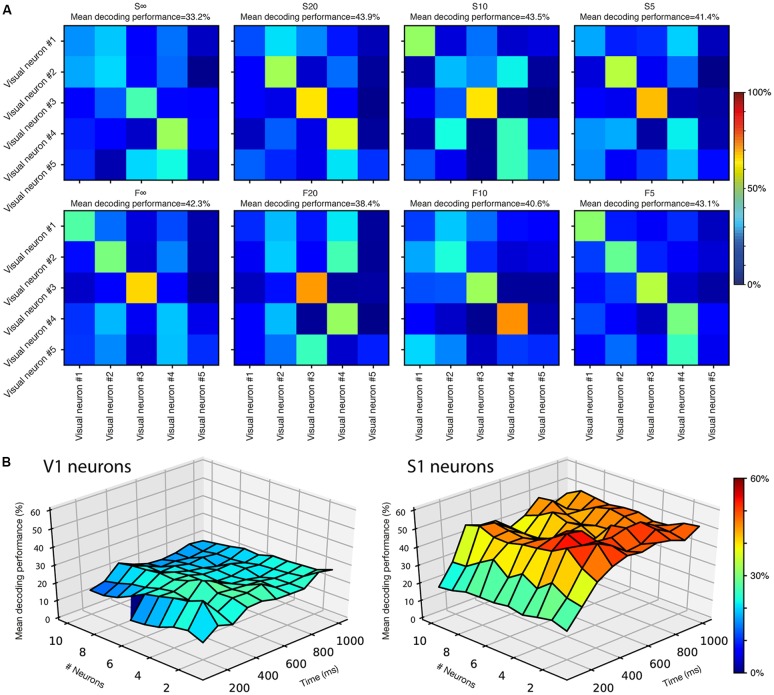
Complementariness of the neuronal responses. **(A)** Decoding analysis for the differences in response patterns for the five top decoding V1 neurons when presented with the same stimulation patterns. One confusion matrix is shown for each stimulation pattern. **(B)** Cooperative decoding analysis for V1 neurons and S1 neurons, respectively. Note that for V1 neurons, the line at 100 ms is interrupted after six neurons, as neurons 7–10 did not provide any responses for this first time window.

### Bootstrapping of Time-Continuous Signals

The continuous responses from a neural recording were recombined or “bootstrapped” by first grouping them by stimulation pattern. *N* unique recorded response sweeps (where *N* as a rule equaled 10, but in the analysis of the EEG state-segmented responses *N* equaled 3) were randomly combined. Each generated response was a unique combination of response sweeps, and each recorded response sweep was present in at least one generated combination. Recorded response sweeps without any spikes were excluded from the bootstrapping procedure and hence the decoding analysis. The sum of each combination of time-continuous response sweeps was stored as a new bootstrapped response. The target was to generate 200 responses for each training or test set. If the number of possible unique combinations for a specific set of responses was below the target, the maximum possible number was used instead. If the number of valid recorded responses fell below the value of *N*, the bootstrapping failed and that part of the data could not be used in the analysis. This condition applied primarily in a few cases of the EEG segmentation analysis.

Note that the above approach is different from methods that rely on comparing the arithmetic means of responses evoked by different stimulation patterns. Assume that a neuronal response to a certain stimulus has more than one type of stable response, as well as superimposed noise. In that case, when comparing the arithmetic means evoked by a number of different stimulation patterns, the arithmetic means would indicate smaller differences between the responses than the actual differences between the underlying distributions. Bootstrapping, which our approach is derived from, in contrast generates a distribution of possible, idealized individual responses. The underlying distribution of responses will determine what kinds of bootstrapped responses that will be generated, and thus also represent a single neuronal response to the stimuli in an idealized manner. When combined with the other components of this analysis, the result will be a graded metric of the neuronal decoding capacity, which takes into account the possibility that the same peripheral input can generate several different responses but that those responses are anyway more different from the responses generated by other inputs.

### Decoding Performance Across Different Time Windows

In addition to the standard time window of 600 ms above, we evaluated the dependency of the decoding on the total time integration window considered. This part of the decoding analysis was performed with 100 ms incremental increase from 100 ms up to 1,000 ms (Figure 6). The statistical difference between neurons in S1 and non-S1 for each integration time window was evaluated with the Mann-Whitney *U* test.

**Figure 5:**
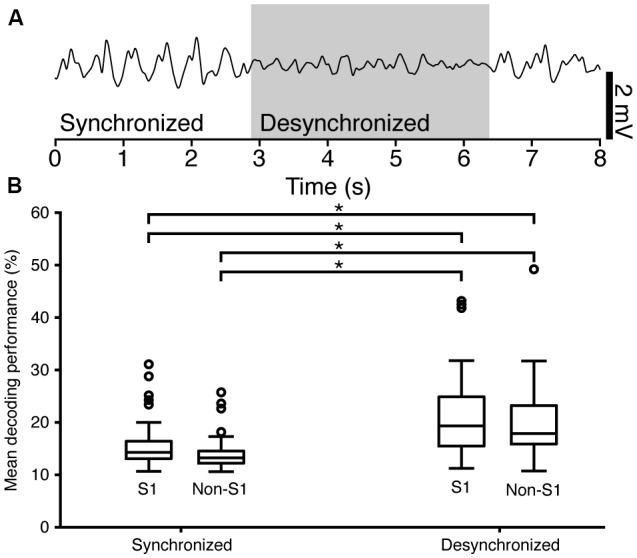
Electroencephalogram (EEG) state segmentation and decoding performance. **(A)** An example of a raw EEG recording, smoothed by a 100 ms moving Hanning window. The segment of the recording that was classified as being desynchronized is indicated by a box in light gray. **(B)** Box-plot of the mean decoding performance for S1 neurons grouped by EEG state (Synchronized *N* = 43; Desynchronized *N* = 26) and for non-S1 neurons, also grouped by EEG state (Synchronized *N* = 24; Desynchronized *N* = 20). Connecting lines with star between sub-groups indicate statistically significant differences as specified in Table 2.

**Figure 6:**
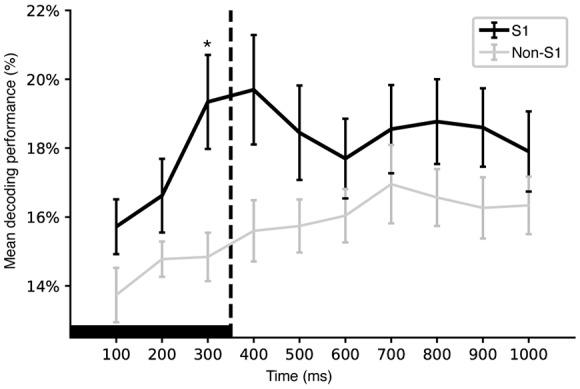
Time evolution of mean decoding performance. Plot of the evolution of the mean decoding performance for individual S1 and non-S1 neurons across integration time windows of increasing duration. The integration time is indicated along the *x*-axis. Whiskers indicate standard error of the mean. A star indicates a statistically significant difference for that time window (*p* < 0.05, Mann Whitney *U* test). Note that the *y*-axis starts at chance level (12.5%). Solid black horizontal bar indicates the duration of the longest stimulation pattern, the dashed line indicates the time of its termination.

#### Decoding Performance Across Different Layers

A common assumption is that neuronal specificity of processing is related to in which layer the neuron is located. To elucidate this question relative to the decoding performance we grouped the neurons according to which layer they were located. This segmentation was done based on their depth from the cortical surface and the following laminar depth boundaries: L1-L2/3: 157 μm, L2/3-L4: 575 μm, L4-L5: 900 μm, L5-L6: 1,411 μm; L6-white matter (WM): 1,973 μm (Narayanan et al., 2017). The neurons were further separated into their respective S1 and non-S1 groups. For each group, the Kruskal-Wallis test was used to test if the distributions of decoding performance in any layer differed from the others.

#### Brain State Segmentation

During each neuronal recording, a parallel ECoG signal was recorded at a sample rate of 1 kHz from the surface electrode placed on the surface of the cortex at the rostral end of the craniectomy (see above). For the brain state segmentation analysis (Figure 5A), the spectral density of the ECoG was calculated with a segment length of 1,000 ms, an overlap of 125 ms and a constant (mean) detrending. The spectral density of Delta, Theta and Alpha bands (0–12 Hz) was summed for each segment and the compound value was used for the remainder of the analysis. An asynchronous segment of ECoG was assumed to occur when the compound spectral density dropped below the compound spectral density median for at least two segments in sequence.

For each recording, every stimulus presentation was classified as occurring either during an asynchronous or a synchronous EEG state, according to the definition above. To be classified as occurring during asynchronous ECoG, the stimulus presentation had to start within a desynchronized segment, and the subtracted time value between the stimulus onset and the end of the desynchronized segment had to exceed 350 ms.

Finally, information content analysis was performed as previously described for spike responses grouped by ECoG state.

## Results

The main scope was to address the question if the spike output of neurons located outside the digit region of the S1 cortex could be used to identify spatiotemporal tactile afferent input patterns applied to the volar side of the left distal digit 2 (Figure 1A). We recorded from a total of 116 neurons distributed across most of the dorsal surface of the neocortex in the anesthetized rat (Figure 1B). Sixty-four neurons were recorded in the parietal and occipital parts of the cortex, including a large fraction (*N* = 51) in the primary visual cortex, V1, according to stereotaxic coordinates (Paxinos and Watson, 2006; Chubykin et al., 2013; Xu et al., 2007). Prefrontal, frontal and lateral temporal cortices were not included due to the more complicated anatomical access. Furthermore, to provide a comparison for the responses of non-S1 neurons (*N* = 64), we recorded many neurons within the primary somatosensory cortex, S1 (*N* = 52). Note that in contrast to our previous study of neuronal decoding of tactile input patterns in S1 (Oddo et al., 2017), in this study only a minority of the S1 neurons were located within the forepaw area of S1. Each animal contributed to 4–10 recording sites. In total, we made recordings from 78 sites. For each recording site, 1–6 neurons were recorded at depths between 292 and 1,446 μm. In addition, we made a separate set of control recordings from 16 neurons in V1 under conditions of reduced audiovisual background (only decoding analysis, see below).

We used both single pulse stimulations to individual skin sites (Figure 1A) and spatiotemporal patterns of skin stimulation across the skin sites to evoke neural responses. All of our recordings were made in the extracellular mode which permitted the recording of sensory-evoked local field potentials (SE-LFP; Figure 1C) through the same electrode that recorded the neuronal units (Figure 1D). SE-LFP responses evoked by the single pulse stimulation were clearly larger in the paw region of S1 than in non-S1 regions (Figure 1C), which suggested that our stimulation generated tactile afferent thalamocortical input with the densest representation located in S1, as expected (Frostig et al., 2008).

We also made a separate analysis of the LFPs evoked by the full stimulation patterns (see below) as they had a longer duration and thereby potentially a higher probability of evoking LFPs in non-S1 regions. The distribution of the differences of the mean number of LFPs in pre- vs. post-trigger periods was not symmetric for S1 intracortical recordings (Wilcoxon signed-rank test; *H* = 12, 566.5, *p* = 5.07e-17; *N* = 52), i.e., indicating a presence of SE-LFPs in S1. But outside S1, we could not find any difference between LFP occurrences in the pre- and post-trigger periods (Wilcoxon signed-rank test; *H* = 45,933.0, *p* = 0.112; *N* = 64). Furthermore, the distribution of the AUC for the LFPs in the pre- and post-trigger periods was not symmetric for S1 (Wilcoxon signed-rank test; *H* = 18, 651.0, *p* = 5.97e-08; *N* = 52), but was symmetric outside S1 (Wilcoxon signed-rank test; *H* = 53, 796.0, *p* = 0.960, *N* = 64). Hence, in agreement with the single pulse stimulation results above, our tactile stimulation patterns neither evoked an increase in LFP frequency nor in LFP AUC in non-S1 regions, despite that the same stimulation did evoke such increases in S1.

### Simple Neuronal Response Measures

For the neuronal spikes, we first tested whether the recorded neurons had any robust response to non-patterned, single pulse electrical stimulation of a single skin site, i.e., the same type of stimulation that we used to quantify the SE-LFPs in Figure 1C. Only eight neurons passed the arbitrarily set, restrictive threshold of requiring the post-trigger spike firing change to exceed 3 SDs from baseline activity to be classified as having a response to this stimulation. All of these neurons were located in S1 (six of eight responses occurred within 20 ms of the onset of the stimulus), which was an expected result. However, using the spatiotemporally patterned stimuli, a total of 39 neurons located both inside (*N* = 26) and outside (*N* = 13) S1 had responses that passed our threshold criterion (+3 SD change relative to baseline) for identifying a response where the response latency time could be calculated. The median response latency time for the S1 neurons was 15–20 ms compared to 35–40 ms for non-S1 neurons (Figure 1E). The difference in the response latency times between the two groups of neurons was statistically significant (two-sided Wilcoxon rank-sum test, *p* = 5.50e-04). Note that the differences in response latencies primarily reflect the intensity of the responses, and do not necessarily indicate their route of mediation. In our data, it is quite possible for a neuron to receive direct thalamocortical input without generating a response that crosses our conservative +3 SD threshold criterion used to identify the latency time. This is, in part, related to the fact that we used relatively weak stimulation of the tactile primary afferents. Using more intense stimulation of tactile afferents, it has previously been shown that direct, short latency thalamocortical responses occur also outside S1 (Zhang et al., 2001) although it is well known that at weaker tactile stimulation intensities response latencies are overall the shortest in S1 (Ferezou et al., 2007). We also explored whether there was a relationship between the response onset latency time and the distance from the digit 2 region of S1 of the recorded neuron. Linear regression showed that such a relationship existed but with a large unexplained residual (*p* = 0.004; *r*2 = 22.3%; *N* = 39), where the latter for example was caused by the presence of V1 neurons with a relatively short response latency time (see Figures 2B,C).

Figure 1F illustrates the intensity of the responses expressed as the number of SDs relative to the baseline. The median value was 9.8 times SD for S1 neurons and 6.4 times SD for non-S1 neurons, with values as high as 148 times SD being observed in S1. Also in this case, there was a significant difference between the S1 and the non-S1 neurons (two-sided Wilcoxon rank sum test; *p* = 0.027; Figure 1F). However, perhaps the most remarkable observation was the presence of robust responses to the tactile afferent activation also in neurons located well outside the S1 region.

### Neuronal Response Patterns and Their Measures

In the remainder of the “Results” section, we analyze the neuronal response patterns generated by spatiotemporal patterns of tactile afferent activation. These stimulation patterns were previously generated by dynamically indenting an artificial fingertip, equipped with four biomorphic sensors, against objects of different shapes (Figure 2A). The shape of the object and the dynamics of the spike generation in the sensors were the bases for the labeling conventions for the stimulation patterns (Figure 2A). These stimulation patterns, which were the same as in the article where this approach was introduced (Oddo et al., 2017), were delivered to the second digit of the contralateral forepaw of the rat where each stimulation site, or channel, corresponded to one of the four sensors of the artificial fingertip (Figure 2A).

Figure 2B illustrates the spike responses of a sample neuron recorded in V1. The raster plots illustrate a quite large spiking variability across the 100 repetitions of each stimulation pattern, which is in contrast to the more regular spiking responses that can be found in some neurons within the paw region of the S1 cortex (e.g., Oddo et al., 2017). Conventional PSTHs illustrated that most of the stimulation patterns nevertheless evoked specific responses in this V1 neuron (Figure 2B) identifiable as differences in the number, onset latency times, amplitudes and widths of the peaks of activation. Note that relatively minor differences between specific stimulation patterns could still result in quite different responses as in the case of S∞ and F∞. In fact, the early response peak of the F∞ stimulation was much greater than for the S∞ stimulation pattern, despite that the only difference between the two inputs at that point in time was that the interval between the two first pulses of the respective patterns differed by 14 ms. For this neuron, conversely, the difference between the responses to the F∞ and the F20 stimulation patterns was minor despite quite large differences between the stimulation patterns. As we have described before Oddo et al. (2017), among S1 neurons it is not uncommon to have clearly separable responses to most stimulation patterns, but that the responses to a specific pattern can sometimes be harder to distinguish from those evoked by another specific pattern. This phenomenon hence occurred also for this V1 neuron, as well as other S1 and non-S1 neurons (not shown). Such unique response profiles form the basis for cooperative decoding between neurons, as we will describe further below.

Whereas the PSTHs hence could identify some consistent differences between responses evoked by different stimulation patterns, PSTHs discard a lot of information in the evoked spike responses by binning the time of occurrence of the spike into a specific interval (i.e., a spike occurring at 11.3 ms would, in this case, be assigned a value of anywhere between 10 and 20 ms). Using KDE, the exact time of occurrence of the spike is given a larger impact, as each spike event is represented as a Gaussian distribution around that time point. Hence, rather than treating a spike response as a discrete event, it is turned into a continuous function. The KDE curve is the sum of all the Gaussian distributions of all the spikes evoked by the stimulation. As shown, after normalization, the KDE curves always fit very closely to the shape of the corresponding PSTHs but have the advantage of a much higher time resolution. Thus, in the example neuron, where the responses to the F∞ and F20 stimulation patterns were quite similar according to the PSTHs, the KDE showed that the single peaks of each response differed in time by more than 5 ms (Figures 2B,C).

To make it possible to quantify the consistency of these responses across repeated presentations of each stimulation pattern, and to compare it with responses evoked by other stimulation patterns, we subdivided the responses into several time windows (Figure 2C). The definition of the time windows used was made on a neuron-by-neuron basis and was designed to depend on the location of the response peaks in the KDE curves for all eight stimulation patterns superimposed (Figure 2C). For example, the relatively small time difference between the first peaks of the F∞ and the F20 responses was captured by this method as the peaks fell partly into different time windows. As different neurons could display different numbers of peaks, a different number of time windows were defined for each neuron. The illustrated V1 neuron had 17 peak-defined time windows (Figure 2C). Across the population of neurons recorded, the number of time windows defined varied from 4 to 27 (15.1 ± 4.4, mean ± SD).

### Decoding Analysis for Individual Neurons

In the comparison of the responses, we considered each of these defined time windows as a dimension for which the metric of each response could be defined. To reduce the sensitivity to spurious spikes occurring in low-intensity responses, we combined the responses in sets of 10 (see “Materials and Methods” section) before analyzing their metrics. Each combined response had a position in high-dimensional space that was defined by its magnitude in each defined time window. Using Euclidean distance calculation against a training set of responses (see “Materials and Methods” section), the nearest neighboring responses could be calculated for each of the 200 combined responses generated for each stimulation pattern. The most common stimulation pattern evoking the responses corresponding to the nine nearest neighboring points in the high-dimensional space was then used to determine if the analyzed response sweep was correctly or incorrectly classified. The accuracy of the classification of all individual response sweeps was summarized in confusion matrices as in Figure 3A. The mean decoding performance was calculated from these confusion matrices using the values in the diagonal (outlined in red in Figure 3A). These values indicated the reliability by which the responses generated by the same stimulation pattern could be separated from the responses generated by the other stimulation patterns. The other boxes in the confusion matrix indicated whether there was any specific stimulation pattern that the tested stimulation pattern was preferentially confused with. Thus, in the illustrated example, the responses generated by the F5 stimulation pattern tended to be classified as being generated by the S5 stimulation pattern, but the reverse was true only to a more limited extent (Figure 3A). Note that the method used here is simpler and more straight-forward than the one we previously used (Oddo et al., 2017), which has the drawback that the values of mean decoding performance reported are not directly comparable.

Figure 3B summarizes the distribution of the mean decoding accuracy across the population of recorded S1 neurons and non-S1 neurons, respectively. For some of the neurons, the KDE curves did not contain peaks of sufficient intensity and number to qualify for this analysis (see “Materials and Methods” section; this applied to 8 of 52 neurons in S1 cortex; 40 of 64 neurons in non-S1 cortex). These neurons are hence not considered in the further analysis made in this article. For the neurons that surpassed this threshold criterion, most neurons in both S1 and non-S1 generated responses that resulted in decoding levels above chance (12.5% for the eight stimulation patters used). The average mean decoding performance for S1 cells was 18.0% (SDs = 6.7%; *N* = 44). The mean decoding performance of these neurons when the stimulation pattern labels for each response was shuffled was 12.5% (mean, SDs = 1.0%), i.e., exactly at chance. For non-S1 cells the mean decoding performance was 15.9% (SDs = 3.54%; *N* = 24) whereas their shuffled control decoding again was at chance level (mean = 12.6%, SDs = 0.8%). We used a two-sided Mann-Whitney *U*-test to compare the distribution of the mean decoding performance for S1 and non-S1 neurons but found no significant difference (*p* = 0.237, *U* = 483; Figures 3B,C). Additionally, we tested if the neurons that we found to have a robust deviation in spike firing intensity to the patterned stimulation (Figure 1F) also had different decoding than the weaker responders. We found that there was a statistical difference (*U* = 247.0, *p* = 0.0046; Mann-Whitney *U*-test). In contrast, we could not find any dependence between recording depth (range 292–1,446 μm) and decoding, as quantified using Pearson’s correlation (All cells ρ = −0.08369, *p* = 0.52858; S1 cells ρ = −0.09000, *p* = 0.59628; Non-S1 cells ρ = 0.00493, *p* = 0.98263). We also grouped the cells according to their cortical subarea and by cortical layer and investigated if there was a relationship to the decoding performance using the Kruskal-Wallis test. For these comparisons between neurons in different lamina, in none of the locations was the *p*-value below 0.05 (Table 1). This suggests that the layer location of the neuron did not have a relationship to the decoding level, which is in line with the findings of our previous investigation in S1 (Oddo et al., 2017).

**Table 1:** Statistical comparison of the decoding performance for neurons in different areas and layer.

Location	Total (*N*)	L2/3 (*N*)	L4 (*N*)	L5 (*N*)	L6 (*N*)	Decoding (mean)	Decoding (SDs)	Kruskal-Wallis test
Non-fpS1	25	2	13	10	0	17%	4.8%	*H* = 0.7, *p* = 0.718
fpS1	12	3	5	4	0	20%	9.0%	*H* = 1.1, *p* = 0.572
M	4	0	0	4	0	16%	2.5%	-
A	5	1	3	1	0	16%	2.9%	*H* = 2.1, *p* = 0.344
V	13	1	5	7	0	16%	3.9%	*H* = 3.8, *p* = 0.146

*Distribution of recordings by cortical location and layer. S1 neurons are subdivided by non-frontpaw area (Non-fpS1) and frontpaw (fpS1) area of the S1. Non-S1 neurons are subdivided by motor (M), associative (A) and visual (V) areas of the cortex. The segmentation of layers is according to the depths defined by Narayanan et al. (2017). Differences in decoding performance between layers for each location were investigated using the Kruskal-Wallis test, except for neurons in the motor areas where the only sampled layer was L5 and no comparison could be made. None of the tests reported a p-value below 0.05*.

We made an additional set of experiments with 16 neurons recorded in the visual cortex in animals with a reduced audiovisual background (depth range: 479–1,324 μm). In this case, 9 of 16 neurons had sufficiently intense peaks of activation in their responses to surpass the defined threshold criterion to be included in the decoding analysis. Their average mean decoding performance was 14.9% (SDs =2.47%; *N* = 9; Shuffled control decoding mean = 12.6%, SDs = 1.0%). Differences in the distributions of the mean decoding performance could not be rejected between S1-, non-S1- and the neurons with reduced audiovisual background (Kruskal-Wallis; *H* = 6.47534, *p* = 0.0393). *Post hoc* analysis with Mann-Whitney *U*-test revealed a difference between the distributions of S1 neurons and the neurons with a reduced audiovisual background (*U* = 372.0, *p* = 0.0178) but no difference between the distributions of the other non-S1 neurons and these neurons (*U* = 205.0, *p* = 0.101).

### Complementary Decoding of Tactile Inputs in Neuron Populations Outside S1

As previously shown for S1 neurons (Oddo et al., 2017), we often noted differences in the temporal response patterns to the same stimulation pattern also in different non-S1 neurons. Such complementary response profiles would suggest that the neurons report specific aspects about the tactile input, which in turn can allow for co-operative decoding where a small population of neurons combined achieves an improved decoding compared to the individual neuron (Oddo et al., 2017).

We first tested the five best-decoding V1 neurons against each other. For each of the eight stimulation patterns, we used the same basic approach as for the single neuron analysis above, but now instead compared the responses of the five different neurons to the same stimulation pattern. The confusion matrices in Figure 4A showed that the responses generated by the different neurons overall were quite well separable from each other. Only a few examples of confusion of the responses between different neurons stood out clearly (Figure 4A). This is a remarkable finding given that the non-S1/V1 neurons individually had relatively noisy responses (Figures 2, 3). The inter-neuron mean decoding performance among these five V1 neurons was 40.8 ± 3.3% across the eight stimulation patterns tested (with a chance level of 20% given by that five neurons were compared). For neurons recorded in the S1 cortex, the five best decoding neurons generated responses with an average mean decoding performance across the eight stimulation patterns of 63.6 ± 7.8% (not shown), i.e., these responses were more systematically or distinctly different from each other than among our V1 neurons. A similar analysis for the V1 neurons with reduced audiovisual background resulted in a mean decoding performance of 39.7 ± 3.39% (chance level was in this case 25% as only the four best V1 neurons out of this more limited population of neurons were included).

We next developed the decoding analysis so that it could take into account the signals generated by multiple neurons, at various response durations, and hence provide a measure of the recorded neurons’ cooperative decoding of the eight stimulation patterns (Figure 4B). Among the V1 population of neurons, the maximum decoding level attained was 32% with four neurons at 400 ms as well as with two neurons at 200 ms. These values were higher than the maximum decoding level of the best V1 neuron (24.8%), and hence there was a cooperativity of the responses generated by the different neurons to improve the decoding of the stimulation patterns. Notably, at longer durations and for a higher number of neurons the cooperative decoding level declined, probably because the amount of noise grew faster than the amount of signal provided by the specific population of V1 neurons we recorded. For S1 neurons, in line with the finding that the across-neuron response specificity (Figure 4A) was higher than for V1 neurons, the maximum cooperative decoding was 56% (compared to 46.8% max for the best individual S1 neuron), attained at 400 ms with both two and four neurons. Unlike the V1 population, the S1 cooperative decoding did not decline as much when more neurons were added, possibly because the signal to noise ratio was more favorable among this population of neurons.

### Decoding Performance in Relation to Brain-State

There are profound differences in EEG during wakefulness, sleep and anesthesia and the EEG state is known to affect cortical neuronal signaling. Desynchronous EEG occurs during active processing in the awake animal (Petersen and Crochet, 2013), but states of desynchronous EEG also occur episodically under general anesthesia. To elucidate the relationship that might be present between the EEG state (here analyzed as desynchronized vs. synchronized ECoG, see Figure 5A) and decoding performance for individual neurons, the responses to the tactile stimuli were subdivided based on the concurrent ECoG state as described in “Materials and Methods” section. This resulted in four combinations of locale (S1 and non-S1) and ECoG state (Synchronized and Desynchronized). For each permutation, the mean decoding performance of the individual neurons was analyzed.

The mean relative amount of time spent in the desynchronized ECoG state was 21% across animals (variance: 0.1%) and the proportion of stimulus presentations that occurred in this state was 26.7%. The median (25th/75th percentile) decoding performance for neurons in S1 in the synchronized state (S1/Synchronized) was 14.6% (13.2/16.8); for S1/Desynchronized 19.8% (15.0/25.1); for non-S1/Synchronized 13.9% (12.2/15.6); for non-S1/Desynchronized 17.6% (15.5/23.4; Figure 5B). Differences in the distributions of the mean decoding performance for the four groups defined by localization (S1 vs. non-S1) and recording state (synchronized or desynchronized ECoG) were statistically significant (Kruskal-Wallis test; *H* = 15.97379, *p* = 0.00115). For *post hoc* analysis of the same cells in different states, a two-tailed Wilcoxon signed-rank test was used. For the analysis across different cells (S1 vs. non-S1) a two-tailed Mann-Whitney *U* test was used. The analysis revealed that there was no significant difference between S1 vs. non-S1 neurons in the same ECoG states (two comparisons). However, for all remaining combinations of location and ECoG state, there were statistically significant differences in the decoding level (Figure 5B and Table 2). The finding that desynchronized EEG state was associated with a higher mean decoding performance suggests that our findings come despite a reduction, or apparent injection of noise, caused by the predominant synchronized EEG state induced by the anesthesia.

**Table 2:** Statistical comparison of the decoding performance in different EEG states.

Comparison	Method	Statistic
Non-S1/Synchronous vs. Non-S1/Asynchronous	Wilcoxon signed-rank test	*p* = 1.3e-11, *T* = 28.0
S1/Synchronous vs. S1/Asynchronous	Wilcoxon signed-rank test	*p* = 4.7e-10, *T* = 5.0
Non-S1/Asynchronous vs. S1/Synchronous	Mann-Whitney *U*	*p* = 3.0e-10, *U* = 2799
Non-S1/Synchronous vs. S1/Asynchronous	Mann-Whitney *U*	*p* = 2.1e-10, *U* = 519

*Results of the statistical tests for each comparison (EEG state/locale). See Figure 5 for EEG state segmentation and pooled neuronal decoding performance in different states and locale*.

### Decoding Performance Across Different Time Windows

We also compared the decoding performance of individual S1 and non-S1 neurons across time windows of different total durations. Figure 6 illustrates the time evolution of the mean decoding performance for S1 and non-S1 neurons. A main difference between S1 and non-S1 neurons appeared to be a higher decoding performance for the earliest time windows although it was only at the 300 ms integration time windows that the S1 neurons performed significantly better at *p* < 0.05 (*U*_[0–0.1]s_ = 219.0, *p*_[0–0.1]s_ = 0.06255; *U*_[0–0.2]s_ = 296.0, *p*_[0–0.2]s_ = 0.46677; *U*_[0–0.3]s_ = 439.0, *p*_[0–0.3]s_ = 0.01810; *U*_[0–0.4]s_ = 464.0, *p*_[0–0.4]s_ = 0.10242; *U*_[0–0.5]s_ = 452.0, *p*_[0–0.5]s_ = 0.48547; *U*_[0–0.6]s_ = 455.0, *p*_[0–0.6]s_ = 0.45654; *U*_[0–0.7]s_ = 486.5, *p*_[0–0.7]s_ = 0.53521; *U*_[0–0.8]s_ = 537.5, *p*_[0–0.8]s_ = 0.32877; *U*_[0–0.9]s_ = 586.0, *p*_[0–0.9]s_ = 0.14346; *U*_[0–1.0]s_ = 536.0, *p*_[0–1.0]s_ = 0.55431).

## Discussion

Regardless of location in the dorsal surface of the neocortex, we could record neurons whose spike output segregated specific spatiotemporal tactile activation patterns. Our study hence extends previous observations of unbounded inputs in the neocortex by showing that such neuronal activation can contain information about the “what” component of the input. Moreover, even in V1, the individual neurons were shown to generate different responses to the same input patterns, which led to that the combined decoding accuracy of a population of non-S1 neurons could become substantially higher than for the best-performing individual non-S1 neuron alone.

### Potential Limitations of the Stimulation Approach

Our approach was motivated as follows: the spatiotemporal patterns of skin sensor activation are the primary means that the nervous system has to identify different skin-object interactions. The high reproducibility of the spatiotemporal patterns of the skin tactile afferent activation of the present approach was needed to address the issue of how well cortical neurons can decode such inputs at the highest possible resolution. This is because the corresponding real-world mechanical skin stimuli are associated with a much higher degree of variability in the patterns of tactile afferent activation (Hayward et al., 2014; Jörntell et al., 2014).

The eight stimulation patterns we used were previously found to be within the same activity range and to show the same envelope of temporal firing modulation as biological tactile afferents under a dynamic mechanical skin indentation (Oddo et al., 2017). As we also noted in this previous study, even though each stimulation site can be expected to activate a low number of tactile afferents in relative synchrony, the input we provided can be expected to have been distributed and processed through multiple layers of neuronal network in the cuneate nucleus, thalamus and neocortical circuitry before it reached the neurons we recorded from. Hence, the measured decoding is bound to reflect at least in part the inherent processing mechanisms of the brain. But there are certainly potential limitations with this approach. First, assuming that the physiological structure of the neocortical circuitry has adapted to the statistical space of naturally occurring spatiotemporal patterns of afferent input (Luczak et al., 2009; Berkes et al., 2011; Okun et al., 2015), it would seem that patterns that are outside this space would be less prone to propagate long distances through the network. But this argument suggests that our stimulation patterns would underestimate the effective network propagation of tactile inputs and is not a problem for our conclusion. Alternatively, the activation of a set of local tactile afferents in synchrony could overrule inhibitory control mechanisms (Renart et al., 2010), which may normally be used to prevent extraneous activation of the circuitry. Thereby, the input could have been made to propagate more effectively or more intensely than what is normally the case. However, even in this scenario, our findings illustrate that the pathways required to propagate tactile information globally across the neocortex do exist. And indeed, in humans, tactile input from digit 2 activates EEG signals widely in the neocortex (Genna et al., 2017). Similarly, the anesthesia used in the present study is expected to dampen the neocortical responsiveness to external stimuli (Constantinople and Bruno, 2011) but would not be expected to open new network pathways since the recruitment order of neocortical neurons to spontaneous brain activity (UP states) and natural stimuli is largely unaffected by anesthesia (Luczak et al., 2009; Luczak and Barthó, 2012). Moreover, as shown in Figure 5, the neuronal decoding increased when the ECoG became desynchronized, which suggest that the anesthesia by inducing synchronized ECoG states injects noise in the spike responses and thus led to an underestimate of the potential neuronal decoding. In awake conditions, without anesthesia, these limitations do not apply. However, in awake conditions, it may instead be difficult to unequivocally identify a recorded cortical response as being generated by the sensory stimulus itself rather than an internally generated signal (Eskandar and Assad, 1999).

### The Decoding Analysis vs. the Organization of Brain Processing

Our decoding analysis built on a quantification of the precision by which the magnitudes of the neuronal spike responses in different time windows could be used to segregate the eight stimulation patterns used. The neuronal circuitry of the brain may well analyze the incoming input in a quite different manner and hence the present analysis can merely indicate that information about the “what” aspect of tactile afferent input patterns exists globally in the neocortical circuitry. How it is used by the brain, and how it contributes to the shaping of behavior is another issue, which should be the scope of future studies.

### Possible Routes of Activity Propagation

Considering the known connectivity, the possibility that tactile information could be propagated globally across the neocortex is well supported. The neocortex contains billions of neocortical neurons with, on average, 7,000 synaptic connections (Pakkenberg et al., 2003). It was estimated by Arbib et al. (1998) that any neuron in the neocortex connects to any other neuron with synaptic linkages involving no more than five neurons on average, a claim that is well supported from the graph theory concept of “small world networks” (Watts and Strogatz, 1998; Bullmore and Sporns, 2009). There is ample substrate for such widespread distribution both in the cortico-cortical but also the cortico-thalamo-cortical (Lübke and Feldmeyer, 2007; Frostig et al., 2008; Sherman, 2016) connectivity, and some support for the involvement of horizontal cortico-cortical connections in propagating unbounded inputs exists (Frostig et al., 2017). Interestingly, it has been argued that most neurons in the neocortex, at least from layer 2 to layer 6 pyramids, are no more than one intercalated neuron, i.e., two synapses, away from the thalamus (Lübke and Feldmeyer, 2007; Wall et al., 2016). This implies that regardless of which neuron is chosen for recording, assuming a synaptic delay of 0.5 ms, any other neuron will only be 2.5 ms away, plus any potential conduction time. Note also that whereas our data indicates that V1 and other non-S1 neurons can decode tactile input, a human study shows that fMRI activity in S1 can be used to decode visual input (Smith and Goodale, 2015). Hence, the spread of unimodal sensory information appears to work in either direction.

### Implications for the Understanding of the Neocortical Mode of Operation

Our findings extend “growing evidence that neurons in primary somatosensory cortex provide essential processing for integrating sensory stimulation from across the hand” (Qi et al., 2016) by suggesting that neurons across the cortex can integrate precise knowledge of the quality of tactile events with their information processing. This notion fits well with the idea that neocortical processing is essentially multisensory (Ghazanfar and Schroeder, 2006), which would form a natural basis for cross-modality disambiguation (Gori et al., 2010). If the brain uses a large number of neurons located outside the S1 cortex, as well as the entire S1 cortex (Figure 1B), in the processing of tactile inputs, it would implicate access to a very large processing capacity. However, since a paw or a hand contains 1000’s of sensors (Johansson and Flanagan, 2009), each of which have a gradable spike output that varies over time during a skin-object interaction, a very large processing capacity may be required. Indeed, the higher the potential processing capacity, the higher the number of interactions that could potentially be identified, which in itself may have evolutionary advantages.

Starting in the ’80s and ’90s, it was suggested that the neocortex was highly plastic. The assumption behind this idea was that there was a strict map-based organization in the neocortex. The pivotal findings were that the cortical maps could change (Buonomano and Merzenich, 1998) given sufficient practice (Siuda-Krzywicka et al., 2016), in functional loss like hand amputation or blindness (Montoya et al., 1998), or even during reversible inactivation of peripheral nerves (Pettit and Schwark, 1993). The implicit assumption has been that such map changes could only be achieved by means of synaptic and/or structural plasticity. Hence, in a strict map-based, or functional localization, view, structural network changes are needed to bring about functional changes.

But the explanatory power of map-based models has been questioned (see “Introduction” section), also recently (Jonas and Kording, 2017), in principle because there is no apparent potential for them to resolve the underlying mechanisms defining brain function. The present findings raise the question of whether the observations of assumed rapid cortical reorganization described above actually only are cases of dynamical use of neocortical circuitry. Dynamical circuitry reuse would imply that the same circuitry components are part of different functional networks depending on the brain state and thereby accessible for different functions across many contexts (Carmena et al., 2003; Elsayed et al., 2016), which would provide for a much higher brain capacity (Spanne and Jörntell, 2015) than in the traditional functional localization view.

## Data Availability

The data is available on https://figshare.com/s/297c1c4e8f5b4c20c037.

## Ethics Statement

All animal experimental procedures in the present study were in accordance with institutional guidelines and approved in advance by the Local Ethics Committee of Lund, Sweden (permit ID M118-13).

## Author Contributions

JE has first authorship, made the patch-clamp recordings, design and the implementation of the analysis. HJ has senior authorship and together with JE, FB and CO designed the experiments. AS, AM, FB and CO contributed to the analysis and discussion of the results. JE, AM, CO and HJ wrote the article.

## Conflict of Interest Statement

The authors declare that the research was conducted in the absence of any commercial or financial relationships that could be construed as a potential conflict of interest.
